# Genome-wide transcriptional analysis of two soybean genotypes under dehydration and rehydration conditions

**DOI:** 10.1186/1471-2164-14-687

**Published:** 2013-10-06

**Authors:** Li M Chen, Xin A Zhou, Wen B Li, Wei Chang, Rong Zhou, Cheng Wang, Ai H Sha, Zhi H Shan, Chan J Zhang, De Z Qiu, Zhong L Yang, Shui L Chen

**Affiliations:** 1 Oil Crops Research Institute of Chinese Academy of Agriculture Sciences, Wuhan 430062, China; 2 Key Laboratory of Oil Crop Biology Ministry of Agriculture, Wuhan 430062, China; 3 Key Laboratory of Soybean Biology in the Chinese Ministry of Education, Northeast Agricultural University, Harbin 150030, China; 4 Division of Soybean Breeding and Seed, Soybean Research & Development Center, CARS (Key Laboratory of Biology and Genetics & Breeding for Soybean in Northeast China, Ministry of Agriculture), Harbin 150030, China

**Keywords:** Soybean, Dehydration, Digital gene expression tag profile, Rehydration, Differentially expressed genes, Quantitative RT-PCR, Transcription factors, Protein kinases, Regulatory proteins

## Abstract

**Background:**

Soybean is an important crop that provides valuable proteins and oils for human use. Because soybean growth and development is extremely sensitive to water deficit, quality and crop yields are severely impacted by drought stress. In the face of limited water resources, drought-responsive genes are therefore of interest. Identification and analysis of dehydration- and rehydration-inducible differentially expressed genes (DEGs) would not only aid elucidation of molecular mechanisms of stress response, but also enable improvement of crop stress tolerance via gene transfer. Using Digital Gene Expression Tag profiling (DGE), a new technique based on Illumina sequencing, we analyzed expression profiles between two soybean genotypes to identify drought-responsive genes.

**Results:**

Two soybean genotypes—drought-tolerant Jindou21 and drought-sensitive Zhongdou33—were subjected to dehydration and rehydration conditions. For analysis of DEGs under dehydration conditions, 20 cDNA libraries were generated from roots and leaves at two different time points under well-watered and dehydration conditions. We also generated eight libraries for analysis under rehydration conditions. Sequencing of the 28 libraries produced 25,000–33,000 unambiguous tags, which were mapped to reference sequences for annotation of expressed genes. Many genes exhibited significant expression differences among the libraries. DEGs in the drought-tolerant genotype were identified by comparison of DEGs among treatments and genotypes. In Jindou21, 518 and 614 genes were differentially expressed under dehydration in leaves and roots, respectively, with 24 identified both in leaves and roots. The main functional categories enriched in these DEGs were metabolic process, response to stresses, plant hormone signal transduction, protein processing, and plant-pathogen interaction pathway; the associated genes primarily encoded transcription factors, protein kinases, and other regulatory proteins. The seven most significantly expressed (|log_2_ ratio| ≥ 8) genes— Glyma15g03920, Glyma05g02470, Glyma15g15010, Glyma05g09070, Glyma06g35630, Glyma08g12590, and Glyma11g16000—are more likely to determine drought stress tolerance. The expression patterns of eight randomly-selected genes were confirmed by quantitative RT-PCR; the results of QRT-PCR analysis agreed with transcriptional profile data for 96 out of 128 (75%) data points.

**Conclusions:**

Many soybean genes were differentially expressed between drought-tolerant and drought-sensitive genotypes. Based on GO functional annotation and pathway enrichment analysis, some of these genes encoded transcription factors, protein kinases, and other regulatory proteins. The seven most significant DEGs are candidates for improving soybean drought tolerance. These findings will be helpful for analysis and elucidation of molecular mechanisms of drought tolerance; they also provide a basis for cultivating new varieties of drought-tolerant soybean.

## Background

Drought stress is one of the major environmental factors adversely influencing plant growth and development [1]. In the face of globally-increasing areas of aridity and semi-aridity, the improvement of plant drought tolerance is crucial [2]. The primary plant response to water deficit is reduction in water content, leading to stomatal closure and/or decreased transpiration. Because stomatal closure in turn leads to reduction in chlorophyll content and CO_2_ assimilation/photosynthetic rates, stomatal status is currently a topic of intense research interest. Early symptoms of water deficit in plants include leaf wilting and rolling and leaf etiolation. Plants adapt to drought by means of various processes, including stress stimulus response, signal perception, signal transduction, and stress-responsive gene expression, with many morphological and physiological changes at molecular and cellular levels providing protection against biotic and abiotic stress damage [3].

Much biological and genetic diversity exists among different plant species with respect to adaptation to drought stress. Many of these stress adaptive mechanisms are not completely understood. Identification of novel drought-responsive genes from drought-tolerant plants and elucidation of their roles in drought adaptation will help improve the tolerance of drought-sensitive plants [4,5].

Drought tolerance is a complex quantitative trait, with many genes induced in response to drought. Drought-responsive genes were initially identified in Arabidopsis using microarray analysis. The products of these genes can be divided into two main classes [6]: 1) functional proteins, such as water channel proteins, detoxification enzymes, late embryogenesis abundant (LEA) proteins, key enzymes for osmolyte biosynthesis (praline, sugars), and proteases [7-9], and 2) regulatory proteins, including transcription factors (TFs), protein kinases (PKs), and proteins associated with phospholipid metabolism and abscisic acid (ABA) biosynthesis.

Production of DNA sequencing reads using the Illumina/Solexa platform is currently widespread [10]. Illumina/Solexa sequencing-based Digital Gene Expression Tag profile (DGE) technology can be used to comprehensively, economically, and rapidly detect changes in gene expression [11]. Because of its high quantitative accuracy, repeatability, and wide detection threshold [12], DGE technology has been widely used to study differentially expressed genes (DEGs) in various fields of biology, including human disease, animal immunization, and plant research [13-15].

Soybean (*Glycine max* [L.] Merr.) is an important crop plant because of its seed protein and oil content. Soybean quality and yield are adversely influenced by drought stress [16,17], which can cause yield losses of approximately 40%. To overcome these limitations, the identification of key drought-responsive genes is needed. With the completion of soybean genome sequencing, the soybean genome has been well characterized [18]. Multiple genes have been annotated using functional genomic methods and their functions tentatively identified.

In this study, a large number of drought-responsive genes differentially expressed between drought-tolerant and drought-sensitive genotypes were identified using an Illumina/Solexa sequencing system. Some of the DEGs uncovered in this study should aid efforts to understand soybean drought tolerance mechanisms.

## Methods

### Preliminary screening of soybean materials under dehydration

To obtain drought-tolerant and drought-sensitive materials, 57 soybean genotypes were collected from different areas of China (Additional file 1). Sterilized seeds were germinated on moistened filter paper for 5–6 d at 28°C and 60% humidity under a 16 h/8 h (light/dark) cycle. The seedlings were then transferred into plastic boxes and cultured hydroponically. Drought treatment was carried out as follows: when the first trifoliolate leaves unfolded, plants to be treated were transferred into plastic boxes without water. They were allowed to dry for 0, 0.5, 1, 2, 4, 6, 8, 10, and 12 h at 28°C at 60% humidity under a 16 h/8 h photoperiod. Control plants were maintained in water under the same conditions. Drought symptoms (leaf wilting and rolling in the top of the canopy) could be readily observed. Wilting was rated on a scale of 0 to 100 (0 = none, 20–40 = slight, 40–60 = moderate, 60–80 = severe, and 100 = plant death) [19]. Further screening was conducted using the related drought-tolerance indices of relative water content (RWC) and relative electrical conductivity (REC). RWC and REC measurements were performed according to published methods [20,21].

### Sample preparation and library construction

**Figure 1:**
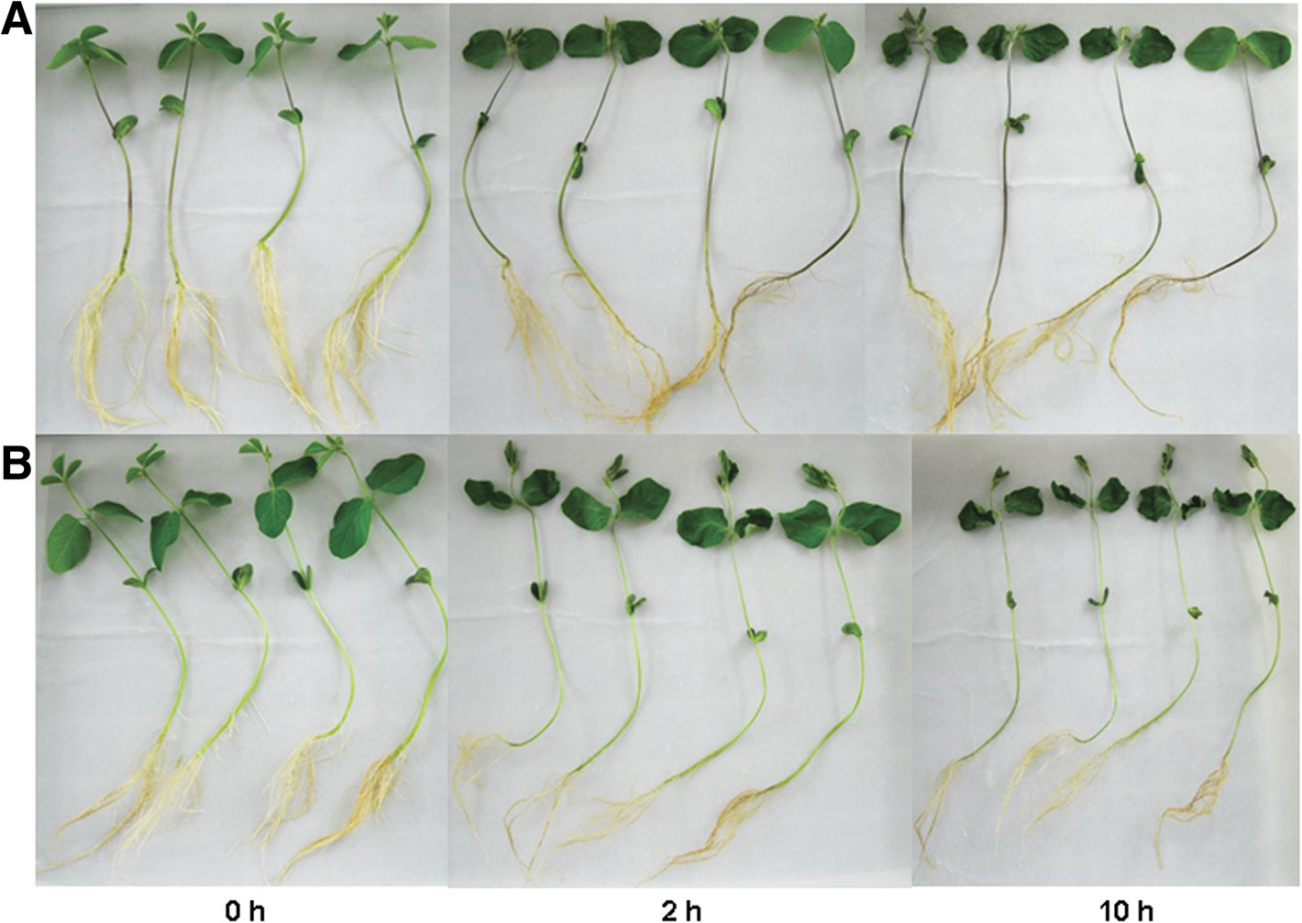
**Phenotypes of two genotypes under 0, 2, and 10 h of dehydration. (A)** Jindou21*.***(B)** Zhongdou33.

Drought-tolerant Jindou21 and drought-sensitive Zhongdou33 were selected for sequencing. Seeds were germinated on filter paper for 5–6 d. Seedlings were transferred to large plastic plates filled with water and grown under greenhouse conditions (28°C, 16 h/8 h photoperiod, 80 μmol m^−2^ s^−1^ photon flux density, and 60% relative humidity). Root and leaf tissues were collected separately when the first trifoliolate soybean leaves unfolded. Drought treatment was carried out as follows [22]: plants were transferred onto filter paper to soak up water, and then allowed to dry for 0, 2, and 10 h at 28°C under a 16 h/8 h photoperiod, 13 μmol m^−2^ s^−1^ photon flux light intensity, and 60% relative humidity. Control plants were maintained in water for 0, 2, and 10 h under the same conditions (Figure 1). In addition, seedlings that had been allowed to dehydrate for 2 h were rehydrated separately for 0.5 h and 2 h. Root and leaf tissues of dehydrated, rehydrated, and control plants were separately collected, with three biological replicates, for sequencing.

Twenty-eight cDNA libraries were generated for sequencing and expression profile analysis: 20 were constructed from dehydrated plants, and 8 were generated from plants undergoing rehydration after a 2-h dehydration treatment (Table 1).

**Table 1:** 28 libraries for sequencing under dehydration and rehydration conditions

**Process**	**Library**			
	JW0L	JW0R	JD2L	JW2L
	Jindou21-water	Jindou21-water	Jindou21-dehydration	Jindou21-water
	0 h-leaves	0 h-roots	2 h-leaves	2 h-leaves
**Dehydration**	JD2R	JW2R	JD10L	JW10L
	Jindou21-dehydration	Jindou21-water	Jindou21-dehydration	Jindou21-water
	2 h-roots	2 h-roots	10 h-leaves	10 h-leaves
	JD10R	JW10R	ZW0L	ZW0R
	Jindou21- dehydration	Jindou21-water	Zhongdou33-water	Zhongdou 33- water
	10 h-roots	10 h-roots	0 h-leaves	0 h-roots
	ZD2L	ZW2L	ZD2R	ZW2R
	Zhongdou33-dehydration	Zhongdou33-water	Zhongdou33-	Zhongdou33-water
	2 h-leaves	2 h-leaves	dehydration 2 h-roots	2 h-roots
	ZD10L	ZW10L	ZD10R	ZW10R
	Zhongdou33-dehydration	Zhongdou33-water 10 h-leaves	Zhongdou33-dehydration	Zhongdou33-water 10 h-roots
	10 h-leaves		10 h-roots	
	JR0.5 L	JR0.5R	JR2L	JR2R
	Jindou21- rehydration	Jindou21- rehydration	Jindou21- rehydration	Jindou21- rehydration
	0.5 h-leaves	0.5 h-roots	2 h-leaves	2 h-roots
**Rehydration after 2-h dehydration**	ZR0.5 L	ZR0.5R	ZR2L	ZR2R
	Zhongdou33-rehydration	Zhongdou33-rehydration	Zhongdou33-rehydration	Zhongdou33-rehydration
	0.5 h-leaves	0.5 h-roots	2 h-leaves	2 h-roots

Each cell lists genotype (Jindou21 or Zhongdou33), treatment (water control, dehydration, or rehydration), treatment duration, and tissue sampled.

### Illumina/Solexa sequencing and clean tag library formation

Sequencing and library formation were performed using an Illumina Gene Expression Sample Prep Kit and a Solexa Sequencing Chip (flow cell), with primary instrumentation consisting of an Illumina Cluster Station and an Illumina HiSeq 2000 System. Raw sequences, including 3' adaptor fragments, low-quality sequences, and several types of impurities, were generated as detailed in Additional file 2. Raw sequences were transformed into clean tags by (1) trimming 3' adaptor sequences from the 49-nt raw reads, and then removing (2) empty reads (reads containing 3' adaptor sequences without tags), (3) low quality tags (tags with unknown sequences ‘N’), (4) tags with lengths of other than 21 nt, and (5) tags with a copy number of one (probably due to sequencing error). In the remainder of this paper, the term ‘total clean tags’ corresponds to the number of clean tags generated, while ‘distinct clean tags’ refers to the number of clean tag types produced. Tag preparation principles and steps are further detailed in Additional file 2.

### Gene expression annotation

A virtual tag library containing all 17-nt + CATG sequences was generated using the SoyBase soybean genomics database (http://www.soybase.org/). All clean tags were mapped to these reference sequences, with only tags having mismatches ≤ 1 bp retained. After filtering out clean tags mapping to reference sequences from multiple genes, the remaining clean tags were designated as unambiguous clean tags. The number of unambiguous clean tags for each gene was calculated and normalized to the number of transcripts per million clean tags (TPM).

### RNA isolation, DNase I treatment, and cDNA synthesis

Total RNA was isolated using Trizol reagent (Invitrogen, USA) and quantified with an Epoch Multi-Volume Spectrophotometer system. Each 6-μg total RNA sample was digested with RNase-free DNase I (Fermentas, Canada) to remove genomic DNA contamination. After DNase I treatment, RNA concentration was checked again; A260/A280 values of all RNA samples used in this study ranged from 1.8 to 2.2 and A260/A230 ratios of all samples were above 2.0. First-strand cDNA synthesis was performed in 20-μl reaction volumes using a DNA Synthesis kit (Promega, USA).

### Housekeeping gene selection and quantitative real-time PCR analysis

Housekeeping genes are used as reference genes to quantify gene expression. Although reference genes should ideally be expressed at a stable level throughout the plant and not be influenced by exogenous treatments [23,24], studies have found that transcription levels of some housekeeping genes vary considerably in response to changes in experimental conditions and across different tissue types [25,26]. For this reason, we selected five housekeeping genes to evaluate levels of gene expression: TUA5, ACT2/7, UBQ10, UKN2, and HDC (Additional file 3). Stability of reference gene expression was analyzed using geNorm software (v3.50). We created a ranking of stability measure values by stepwise exclusion of the least stable housekeeping gene, and then used the relatively most stable housekeeping gene to normalize expression levels of selected genes [27].

Primers were designed using Primer 5.0 software. Expression levels for all candidate genes were computed based on the stable expression level of the reference gene. Quantitative real-time PCR (QRT-PCR) was performed in 96-well plates on a Bio-Rad iQ5 Real-Time PCR Detection system using SuperReal PreMix (SYBR Green) reagents (Tiangen, China). Reactions were carried out in 10-μl volumes containing 0.3 μM final concentrations of each primer. The QRT-PCR thermal profile consisted of 95°C for 15 min, followed by 40 cycles of 95°C for 10 s and 60°C for 32 s. Dissociation curves were obtained from a thermal melting profile generated under a final PCR cycle of 95°C for 30 s followed by a constant increase in temperature from 60°C to 95°C. Threshold values were empirically determined based on the observed linear amplification phase of all primer sets. Sample cycle threshold (ct) values were standardized for each template based on reference gene control primer reaction, and the 2^–∆∆CT^ method was used to analyze relative changes in gene expression [28]. Three replicate reactions per sample were used to ensure statistical credibility.

## Results

### Screening of soybean genotypes for drought tolerance

**Figure 2:**
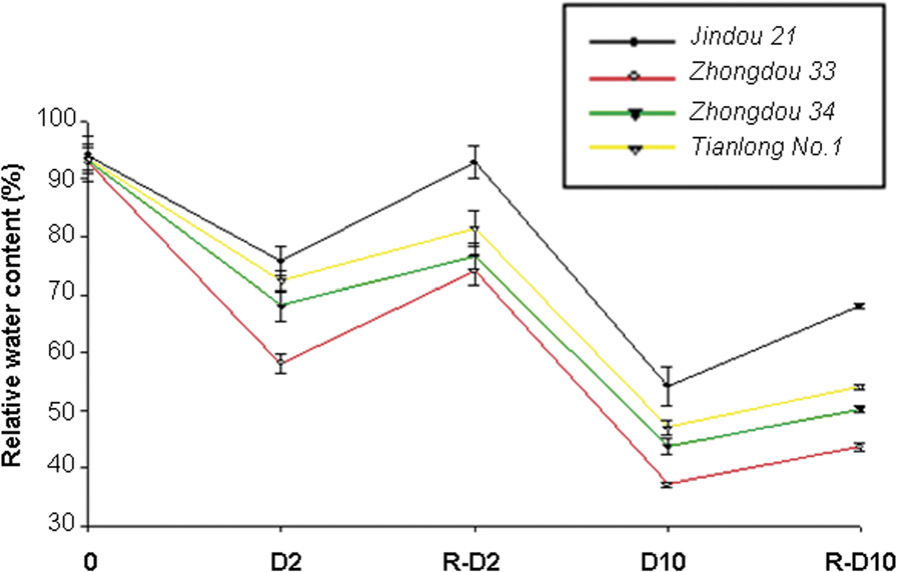
**Relative water content of four genotypes under dehydration for 0, 2, and 10 h and under rehydration.** D2 = 2 h dehydration; R-D2 = rehydration after 2 h dehydration; D10 = 10 h dehydration; R-D10 = rehydration after 10 h dehydration.

**Figure 3:**
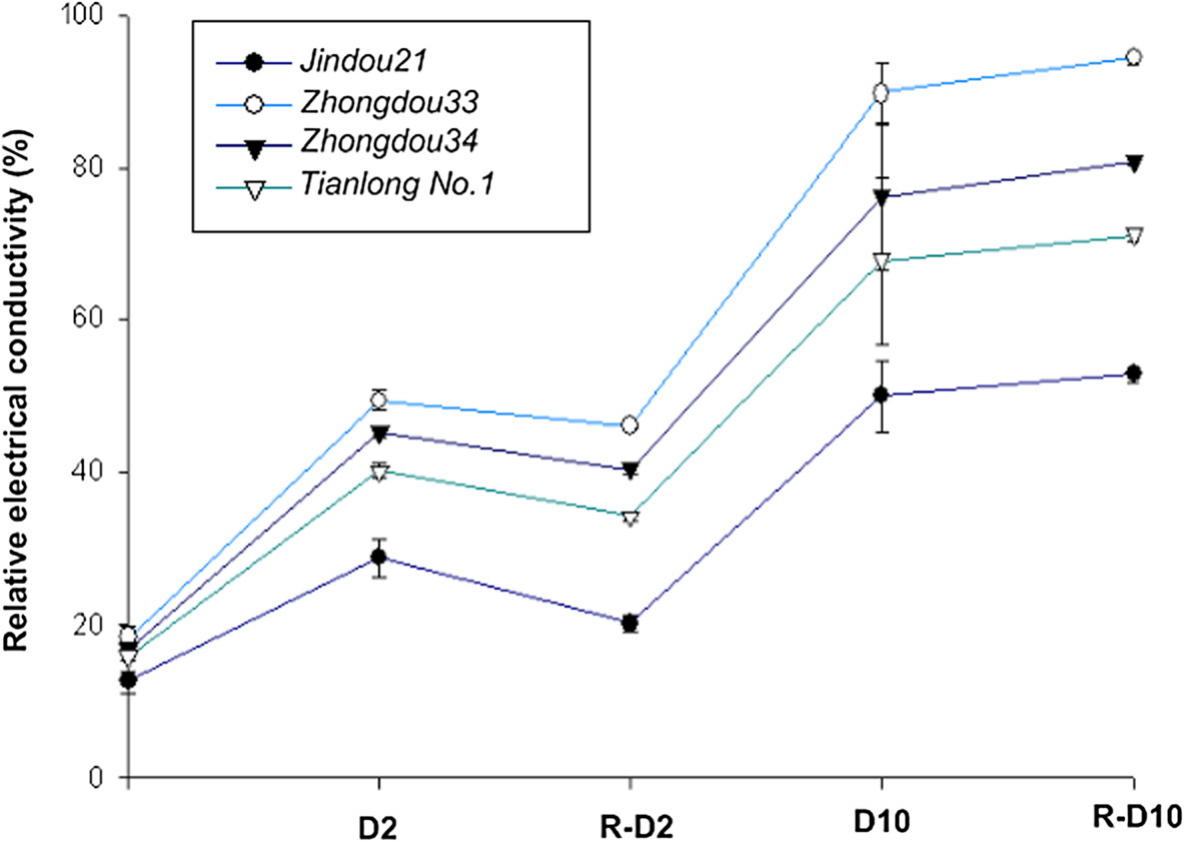
**Relative electrical conductivity of four genotypes under dehydration for 0, 2, and 10 h and under rehydration.** D2 = 2 h dehydration; R-D2 = rehydration after 2 h dehydration; D10 = 10 h dehydration; R-D10 = rehydration after 10 h dehydration.

To identify soybean genotypes with drought tolerance, 57 genotypes were screened at the seedling stage under water-deficit conditions (Additional file 1). Based on preliminary screening experiments, we selected two drought-tolerant genotypes (Jindou21 and Tianlong1) and two drought-sensitive genotypes (Zhongdou33 and Zhongdou34). Further screening experiments revealed significant differences in RWCs and RECs among the four soybean genotypes under dehydration and rehydration conditions. RWCs of all four genotypes declined during dehydration and increased after rehydration, but the RWC of Jindou21 was always higher than that of the other genotypes under drought stress and quickly recovered to control levels after 2 h of rehydration (Figure 2). REC measurements indicated that RECs of all four genotypes increased under dehydration. The extent of increase was lowest in Jindou21 and highest in Zhongdou33, which had the poorest recovery after 2 h dehydration. RECs of all four genotypes were elevated during rehydration following 10 h dehydration (Figure 3), indicating that prolonged dehydration may have damaged the seedling leaves. Based on these results, we choose the drought-tolerant genotype Jindou21 and the drought-sensitive genotype Zhongdou33 for DGE.

### Illumina/Solexa sequencing evaluation

In this study, an Illumina/Solexa system was used for tag sequencing analysis. Twenty-eight samples from Jindou21 and Zhongdou33 were prepared for sequencing: 20 samples were obtained from 0, 2, and 10 h dehydration treatments and 8 samples were derived from 0.5 h and 2 h rehydration treatments. After identifying DEGs between control (water) and dehydration treatments, DEGs between the two genotypes were generated. The number of raw sequence tags generated from each of the 28 libraries ranged from 5.8 to 6.2 million, and the number of raw tags producing distinct sequences ranged from 0.3 to 0.8 million (Additional file 4). To assess Illumina/Solexa sequence quality, a sequencing saturation analysis was performed to check whether the number of detected genes increased with increasing amounts of sequence data. After generation of about three million tags, the number of detected genes barely continued to increase (Additional file 5); at this point, the library was saturated and contained sufficient information for gene expression analysis.

After trimming and filtering the raw sequence data, there were many distinct tags generated from the 28 libraries. The distribution of the various tag abundance categories between total and distinct tag counts was consistent across all libraries. Matching tags to genes is necessary for sequence prediction and determination of molecular mechanisms underlying gene expression. When clean tags were BLAST-searched against available genome data (http://phytozome.net/), more than 75% mapped to known genes. The number of tag-mapped genes represented in each library ranged from 30,155 to 29,706 (Additional file 4).

### Identification of DEGs between the two treatments

**Figure 4:**
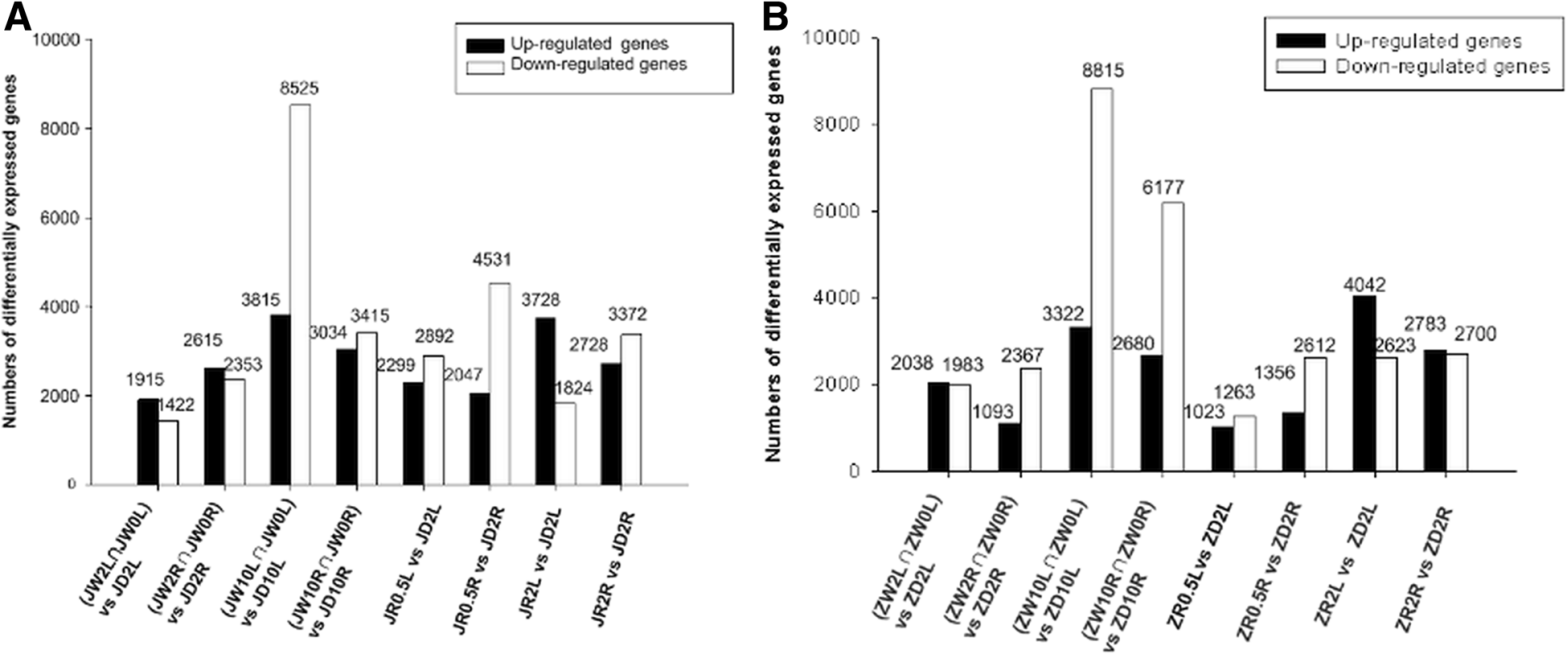
**Number of DEGs in two genotypes under dehydration and rehydration treatments. (A)** Number of up- or down-regulated genes in the drought-tolerant genotype. **(B)** Number of up- or down-regulated genes in the drought-sensitive genotype.

**Figure 5:**
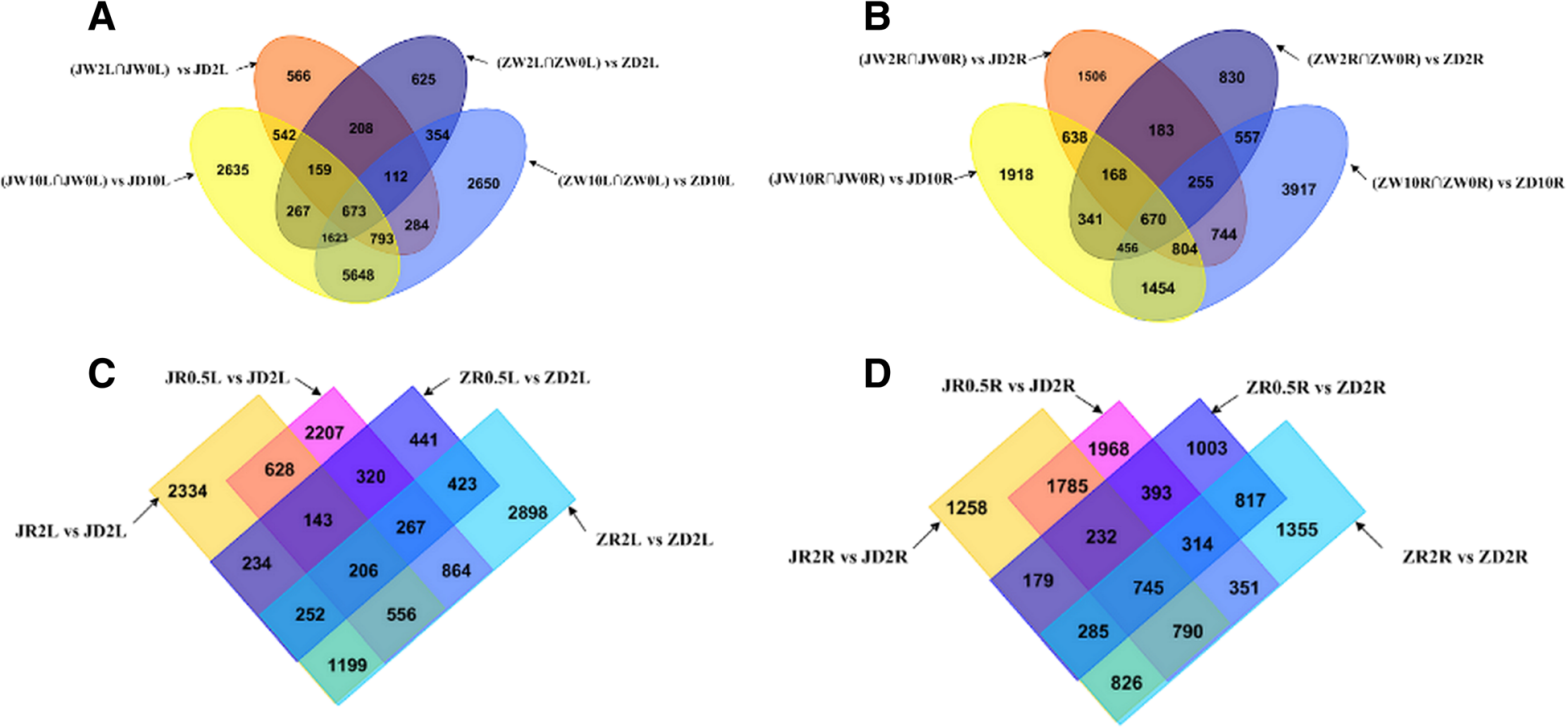
**Venn diagram of DEGs in two genotypes. (A)** Venn diagram of DEGs in leaves under dehydration. **(B)** Venn diagram of DEGs in roots under dehydration. **(C)** Venn diagram of DEGs in leaves under rehydration. **(D)** Venn diagram of DEGs in roots under rehydration.

To judge the significance of differences in expressed genes, we used three criteria: FDR ≤ 0.001, |log_2_ ratio| ≥ 1, and *P*-value < 0.01. In this study, many genes were differentially expressed between control (water) and dehydration conditions, and between dehydration and rehydration treatments. For example, in the drought-tolerant genotype Jindou21, there were 3,337 DEGs between (JW2L∩JW0L) vs. JD2L, 4,968 DEGs between (JW2R∩JW0R) vs. JD2R, 12,340 DEGs between (JW10L∩JW0L) vs. JD10L, and 6,449 DEGs between (JW10R∩JW0R) vs. JD10R. In the drought-sensitive genotype Zhongdou33, 4,021 DEGs were detected between (ZW2L∩ZW0L) vs. ZD2L, with 3,460 DEGs observed between (ZW2R∩ZW0R) vs. ZD2R, 12,137 DEGs between (ZW10L∩ZW0L) vs. ZD10L, and 8,857 DEGs between (ZW10R∩ZW0R) vs. ZD10R. There were also many DEGs between the two treatments. In the drought-tolerant genotype, there were 5,191 DEGs observed between JR0.5 L vs. JD2L, 6,578 DEGs between JR0.5R vs. JD2R, 5,552 DEGs between JR2L vs. JD2L, and 6,100 DEGs between JR2R vs. JD2R; 2,286 DEGs were found between ZR0.5 L vs. ZD2L, 3,968 DEGs between ZR0.5R vs. ZD2R, 6,665 DEGs between ZR2L vs. ZD2L, and 5,483 DEGs between ZR2R vs. ZD2R in the drought-sensitive genotype (Figure 4). A Venn diagram illustrating the division of DEGs into different groups according to dehydration time, expression site, and rehydration process is shown in Figure 5.

### Further analysis of DEGs between the two genotypes

Based on results observed between treatments, differences between the two genotypes were further compared with a focus on DEGs found only in the drought-tolerant genotype. Under dehydration, a number of genes were expressed only in the drought-tolerant genotype: 518 in leaves (Additional file 6), 614 in roots (Additional file 7), and 24 in both leaves and roots. Although some of these genes could be assigned putative functions, the functions of most were unknown. In Jindou21 under rehydration conditions, 563 genes were expressed only in leaves (Additional file 8), 1,720 in roots (Additional file 9), and 65 both in leaves and roots.

### Gene Ontology functional enrichment analysis of DEGs in the drought-tolerant genotype

GO (Gene Ontology), an internationally standardized gene function classification system, is widely used for gene functional annotation and enrichment analysis. GO encompasses three domains: cellular component, biological process, and molecular function. The basic GO unit is the GO term. Every GO term belongs to a particular category. In DGE analysis, GO functional enrichment analysis is performed using hypergeometric testing to map all DEGs to terms in the GO database (http://www.geneontology.org/). To identify significantly enriched GO terms, we calculated the probability (*P*) of a particular GO term being significantly enriched in a given set of genes according to the following formula:

$$Ρ=1-\sum_{i=0}^{m=1}\frac{\left(\frac{M}{i}\right)\left(\frac{N-M}{n-i}\right)}{\left(\frac{N}{n}\right)}$$

where *N* is the total number of genes, *n* is the number of DEGs in *N*, *M* is the total number of genes annotated with the particular GO term, and *m* is the number of DEGs in *M*. GO terms with Bonferroni-corrected *P*-values ≤ 0.05 were defined as significantly enriched in DEGs.

**Figure 6:**
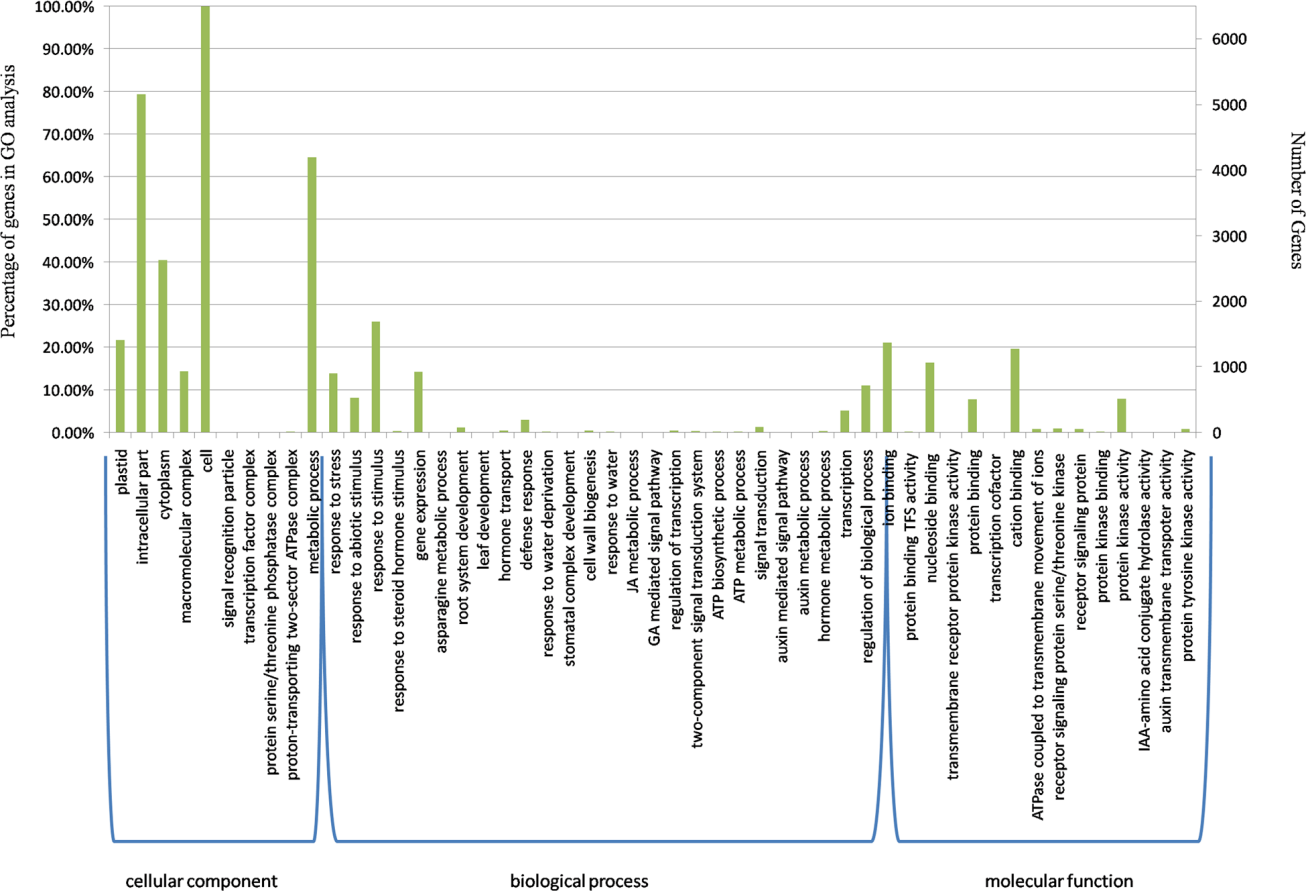
**Histogram of gene ontology classifications.** The three GO domains—biological process, cellular components, and molecular function—are shown. Left and right y-axes indicate gene category frequencies for each domain and the number of genes in each category, respectively.

In our study, 6,582 DEGs were divided into 51 functional groups (Figure 6). With respect to cellular components, most DEGs were involved in the categories of cell structure (GO: 0005623), intracellular parts (GO: 0044424), and cytoplasm (GO: 0005737). Under biological process, 68.2% of DEGs were found to be involved in metabolic processes (GO: 0008152), with 385 DEGs encoding products involved in carbohydrate metabolic processes (GO: 0005975), 318 encoding products involved in lipid metabolic processes (GO: 0006629), 253 having products involved in amino acid metabolic processes (GO: 0006520), and 5 encoding products involved in energy reserve metabolic processes (GO: 0006112). There were 896 DEGs associated with response to stress (GO: 0080134), including response to water deprivation (GO: 0009414), salt stress (GO: 0009651), temperature stimulus (cold or heat) (GO: 0009266), osmotic stress (GO: 0006970), abscisic acid stimulus (GO: 0009737), and metal ions (GO: 0010038). In addition, DEGs were identified in root and leaf systems, including 79 DEGs related to tap and lateral root development (GO: 0048364) and 18 DEGs associated with leaf development (GO: 0048366), of which 10 were involved in stomatal movement (GO: 0010118). In regard to molecular function, many DEGs were found encoding ion binding proteins (GO: 0043167) and receptor signaling proteins (GO: 0004702), with the majority involved in transcription factor (GO: 0003712) activities and protein kinase activities (GO: 0004672) (Additional file 10).

### Pathway enrichment analysis of DEGs in the drought-tolerant genotype

Pathway enrichment analysis is an effective method for elucidation of DEG biological functions. Pathway-based analysis can aid identification of significantly enriched metabolic pathways and signal transduction pathways in DEGs through comparison with their whole-genome backgrounds [29]. The formula used for this calculation was essentially identical to that used in the GO analysis, with pathways having a *Q*-value ≤ 0.05 defined as those with significantly DEGs.

**Figure 7:**
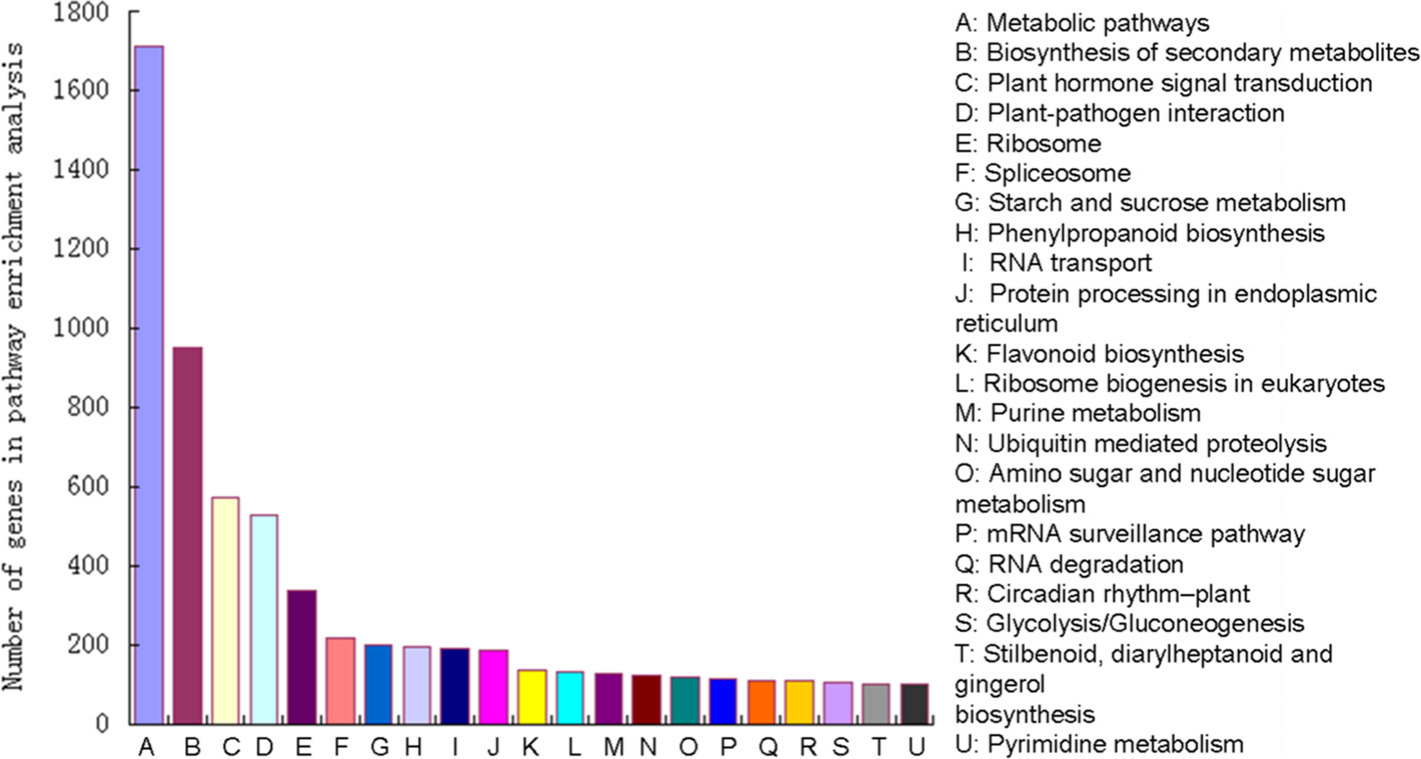
**Results of KEGG pathway enrichment analyses of DEGs for 21 main KEGG pathways.** X- and y-axes represent pathway categories and the number of genes in each pathway, respectively.

KEGG is the major public pathway-related database. In our study, DEGs in the drought-tolerant genotype were associated with 21 KEGG pathways (Figure 7). The pathways with the greatest numbers of unique sequences were metabolic pathways (1,712 members) (ko01100), which are large complexes associated with metabolic processes such as biosynthesis of secondary metabolites and carbohydrate, lipid, and amino acid metabolism [30-33]. Other pathways, such as plant hormone signal transduction (573 members) (ko04075), plant-pathogen interaction (529 members) (ko04626), and protein processing in endoplasmic reticulum (187 members) (ko04141), were also main enrichment pathways [34-37] (Additional file 11). We believe that these pathways may play an important role in plant response to dehydration and rehydration conditions.

GO functional annotation and pathway enrichment analysis of DEGs in the drought-tolerant genotype revealed that the DEGs involved in the most highly enriched biological processes and pathways (Additional files 10 and 11) primarily encoded transcription factors, protein kinases, and other regulatory proteins. These DEGs were more likely to determine drought tolerance than those in the drought-sensitive genotype.

### DEGs encoding transcription factors in the drought-tolerant genotype

To analyze regulatory mechanisms of drought-responsive genes in the drought-tolerant genotype, 37 genes encoding transcription factors (TFs) were identified from Jindou21 leaves and roots (Table 2). These genes could be divided into six major groups. The first group contained seven auxin response factor (ARF) genes. Two of these genes (Glyma14g40540.1 and Glyma13g17270.2), identified in leaves, were suppressed under dehydration. The remaining five genes were identified in roots, four of which (Glyma07g03840.1, Glyma01g33420.1, Glyma07g17810.2, and Glyma13g29320.2) were induced under dehydration. The second group comprised five genes encoding ethylene-responsive factors (ERFs), with four of them (Glyma17g15480.1, Glyma05g05180.1, Glyma13g30990.1, and Glyma18g48740.1) induced under water deficit conditions. The third group was made up of MYB TF family genes. Four (Glyma14g06750.1, Glyma01g01190.1, Glyma11g05550.1, and Glyma02g00820.1) of the five genes were down-regulated under dehydration. The fourth group constituted four zinc-finger protein family genes (Glyma20g23550.1, Glyma06g04410.1, Glyma13g01290.1, and Glyma10g35940.1), two of which were up-regulated and two of which were down-regulated. The fifth group was composed of ring-finger family genes; one member (Glyma20g26780.1) was induced by dehydration in leaves, and two (Glyma04g07980.1 and Glyma13g20210.1) were suppressed in roots. The sixth group included three HD-ZIP family genes (Glyma02g02630.1, Glyma06g20230.1, and Glyma09g39360.1) identified only in roots. Under dehydration conditions, two of these were down-regulated and one was up-regulated. The remaining TF genes encoded heat shock factors (HSFs) (Glyma13g29760.1 and Glyma11g14950.1), members of families such as helix-loop-helix (bHLH), (Glyma13g39650.1 and Glyma15g18580.1), AP2/EREBP (Glyma14g34590.1 and Glyma10g34760.1), and bZIP (Glyma10g01640.1 and Glyma09g38300.1), and other types, including NAC domain protein (Glyma20g31210.2) and Dof6 (Glyma13g31560.1).

**Table 2:** Transcription factors responsive to dehydration only in the drought-tolerant genotype

**Transcription factors (TFs)**			
**Gene**	**Jindou21 leaves (log**_**2**_**ratio)**	**Jindou21 roots (log**_**2**_**ratio)**	**Annotation**
**Auxin-related protein**			
Glyma14g40540.1	−4.32		Auxin response factor(ARF)
Glyma13g17270.2	−2.42		Auxin response factor(ARF)
Glyma04g43150.1		−1.56	Auxin transporter-like protein 3
Glyma07g03840.1		1.84	Auxin-induced protein AUX22
Glyma01g33420.1		1.56	Auxin-induced protein X10A
Glyma07g17810.2		1.26	AUX1-like auxin influx carrier protein
Glyma13g29320.2		2.35	ARF domain class transcription factor
**Ethylene–responsive proteins**			
Glyma17g15480.1	3.31		Ethylene transcription factor 1A
Glyma05g05180.1	1.83		ethylene-responsive transcription factor 1A
Glyma13g30990.1	1.03		ethylene-responsive element binding factor 4
Glyma18g48740.1		1.81	ethylene-responsive element binding protein 1
Glyma15g08360.1		−2.25	ethylene responsive transcription factor 12
**MYB transcription factor family**			
Glyma14g06750.1	−1.76		MYB transcription factor MYB75
Glyma01g01190.1	−1.45		GAMYB-binding protein
Glyma11g05550.1		−2.05	MYB transcription factor MYB50
Glyma15g03920.1		8.12	MYB transcription factor MYB82
Glyma02g00820.1		−1.89	GmMYB29A2
**zinc finger protein**			
Glyma20g23550.1	1.198624		zinc finger protein
Glyma06g04410.1	−1.65		zinc finger protein
Glyma13g01290.1		3.29	CONSTANS-like zinc finger protein
Glyma10g35940.1	−7.94		C2-H2 zinc finger protein
**Ring-H2 protein**			
Glyma20g26780.1	1.12		RING-H_2_ finger protein
Glyma04g07980.1		−1.03	ring finger protein
Glyma13g20210.1		−1.64	RING-finger protein
**HD-ZIP family**			
Glyma02g02630.1		1.05	homeodomain-leucine zipper protein 57
Glyma06g20230.1		2.81	HD-ZIP I protein
Glyma09g39360.1		−1.99	Homeodomain-related
**Heat shock protein**			
Glyma13g29760.1	1.50		heat shock transcription factor
Glyma11g14950.1		1.16	heat shock cognate protein 1
**AP2/EREBP family**			
Glyma14g34590.1		1.86	DREB2
Glyma10g34760.1		−4.07	RAV-like DNA-binding protein
**Helix-loop-helix protein**			
Glyma13g39650.1		7.82	helix-loop-helix protein BHLH21
Glyma15g18580.1		−1.91	Helix-loop-helix DNA-binding
**bZIP transcription factor family**			
Glyma10g01640.1	1.70		bZIP transcription factor bZIP59
Glyma09g38300.1	−7.94		leucine zipper-ef-hand containing transmembrane protein
**NAC family**			
Glyma20g31210.2	2.15		NAC domain protein
**Dof6 family**			
Glyma13g31560.1		−1.20	Dof6 transcription factor

Note: Genes with FDR ≤ 0.001, |log_2_ ratio| ≥1 and *P*-value < 0.01 (**) were identified as differentially expressed genes. Significance at 0.01 level is marked with **.

### DEGs encoding protein kinases (PKs) in the tolerant genotype

To better understand signal transduction processes in soybean under dehydration, 19 DEGs encoding protein kinases were identified in the tolerant genotype (Table 3). Of seven DEGs encoding S/T PKs (Glyma20g38980.1, Glyma13g43580.2, Glyma05g07580.1, Glyma17g12620.1, Glyma03g29450.1, Glyma09g33120.1, and Glyma15g07820.2), three were up-regulated and four were down-regulated under dehydration. Three genes (Glyma05g37260.1, Glyma16g03870.1, and Glyma11g24410.1) encoding CDPKs were down-regulated, and one (Glyma03g29450.1) was up-regulated. Similarly, three genes (Glyma05g02470.1, Glyma05g31120.1, and Glyma20g27580.1) encoding RPKs were suppressed and one gene (Glyma17g32780.1) was induced under dehydration. Only one gene (Glyma08g16670.1) encoding MAPKs was identified in the tolerant genotype. The remaining DEGs (Glyma01g00790.1, Glyma16g06940.1, and Glyma17g08270.1) encoded three other PKs.

**Table 3:** Protein kinases responsive to dehydration only in the drought-tolerant genotype

**Protein kinase family (PKs)**			
**Gene**	**Jindou21 leaves (log**_**2**_**ratio)**	**Jindou21 roots (log**_**2**_**ratio)**	**Annotation**
**S/T PKs**			
Glyma20g38980.1	1.12		serine/threonine protein kinase
Glyma13g43580.2	−2.28		serine/threonine-protein kinase PBS1
Glyma05g07580.1	−2.04		serine/threonine protein kinase
Glyma17g12620.1		−2.23	serine/threonine protein kinase
Glyma03g29450.1		1.37	serine-threonine protein kinase
Glyma09g33120.1		−1.79	serine/threonine-protein kinase cx32
Glyma15g07820.2		1.41	serine/threonine protein kinase
**CDPKs**			
Glyma05g37260.1	−1.69		calcium-dependent protein kinase
Glyma03g29450.1	1.37		calcium-dependent protein kinase isoform 2
Glyma16g03870.1		−1.82	calmodulin-binding receptor-like cytoplasmic kinase
Glyma11g24410.1		−1.23	calmodulin-binding receptor-like cytoplasmic kinase
**RPKs**			
Glyma05g02470.1		−8.23	receptor-like protein kinase
Glyma05g31120.1		−1.26	Somatic embryogenesis receptor kinase-like protein 3
Glyma17g32780.1	2.84		stress-induced receptor-like kinase
Glyma20g27580.1		−1.51	receptor-like protein kinase homolog RK20-1
**PKs**			
Glyma01g00790.1		−1.32	leucine-rich repeat protein kinase
Glyma16g06940.1		1.16	leucine-rich repeat family protein / protein kinase family protein
Glyma17g08270.1	−1.83		protein kinase
**MAPKKK**			
Glyma08g16670.1	−1.47		mitogen-activated kinase kinase kinase alpha

Genes with FDR ≤ 0.001, |log_2_ ratio| ≥ 1 and *P*-value < 0.01 (**) were identified as differentially expressed genes. Significance at 0.01 level is marked with **.

### DEGs encoding other regulatory proteins in the tolerant genotype

Twenty-four genes encoding other drought-responsive proteins were also identified in Jindou21 under dehydration (Table 4). Among them, one gene (Glyma20g30720.1) encoded abscisic stress ripening-like protein and another (Glyma11g12060.1) encoded salt tolerance-like protein. Four genes (Glyma17g37480.1, Glyma14g13480.2, Glyma14g37440.1, and Glyma11g27720.2) encoded two-component systems [38]; two of these genes were down-regulated in leaves, and two were up-regulated in roots. Genes for seven (Glyma20g38610.1, Glyma20g30100.2, Glyma20g30100.2, Glyma15g15010.1, Glyma02g29020.1, Glyma20g27660.1, and Glyma07g27370.1) ATP-binding proteins, three cytochrome P450s (Glyma05g09070.1, Glyma18g03210.1, and Glyma08g20690.1), and two trypsin-inhibitor-like proteins (Glyma09g29310.1 and Glyma09g29330.1) were identified and found to be down-regulated under dehydration. Increased expression was observed for two genes (Glyma06g41290.1 and Glyma06g47780.1) encoding TIR-NBS-LRR-type disease-resistance proteins. In addition, four genes encoding other types of proteins were identified (Glyma01g04470.2, Glyma07g35110.1, Glyma06g35630.1, and Glyma13g07110.1); one was down-regulated in leaves and the others were up-regulated.

**Table 4:** Other regulatory proteins responsive to dehydration only in the drought-tolerant genotype

**Other regulatory proteins**			
**Gene**	**Jindou21 leaves (log**_**2**_**ratio)**	**Jindou21 roots (log**_**2**_**ratio)**	**Annotation**
**Abscisic acid protein**			
Glyma20g30720.1		4.38	abscisic stress ripening-like protein
**Salt stress-responsive protein**			
Glyma11g12060.1		2.19	salt tolerance-like protein
**two-component systems**			
Glyma17g37480.1	−2.02		histone H3.2
Glyma14g13480.2	−2.74		aspartate aminotransferase
Glyma14g37440.1		7.92	asparagine synthetase 2
Glyma11g27720.2		1.05	asparagine synthetase 2
**ATP binding proteins**			
Glyma20g38610.1	−2.23		ATP-binding cassette transporter
Glyma20g30100.2	−1.37		ATP binding protein
Glyma12g04490.1	−1.02		ATP binding protein
Glyma15g15010.1	−10.73		ATP citrate lyase alpha subunit
Glyma02g29020.1		−1.34	ATP binding protein
Glyma20g27660.1		−1.37	ATP binding protein
Glyma07g27370.1		−2.30	ATP binding protein
**cytochrome P450s**			
Glyma05g09070.1	−10.41		cytochrome P450, putative
Glyma18g03210.1	−3.44		cytochrome P450 monooxygenase CYP90A15
Glyma08g20690.1		−1.43	cytochrome P450 enzyme
**TIR-NBS-LRRs**			
Glyma06g41290.1	7.94		TIR-NBS-LRR type disease resistance protein
Glyma06g47780.1		9.07	LRR protein-related protein
**trypsin-inhibitor-like proteins**			
Glyma09g29310.1		−4.08	pathogen-inducible trypsin-inhibitor-like protein
Glyma09g29330.1		−2.39	pathogen-inducible trypsin-inhibitor-like protein
**Other type regulated proteins**			
Glyma01g04470.2		1.41	pyruvate dehydrogenase alpha subunit
Glyma07g35110.1	1.19		pyruvate kinase
Glyma06g35630.1	−11.44		tyrosine aminotransferase
Glyma13g07110.1		2.80	proline dehydrogenase

Genes with FDR ≤ 0.001, |log_2_ ratio| ≥ 1, and *P*-value < 0.01 (**) were identified as differentially expressed genes. Significance at 0.01 level is marked with **.

### Cluster analysis of DEG expression patterns

**Figure 8:**
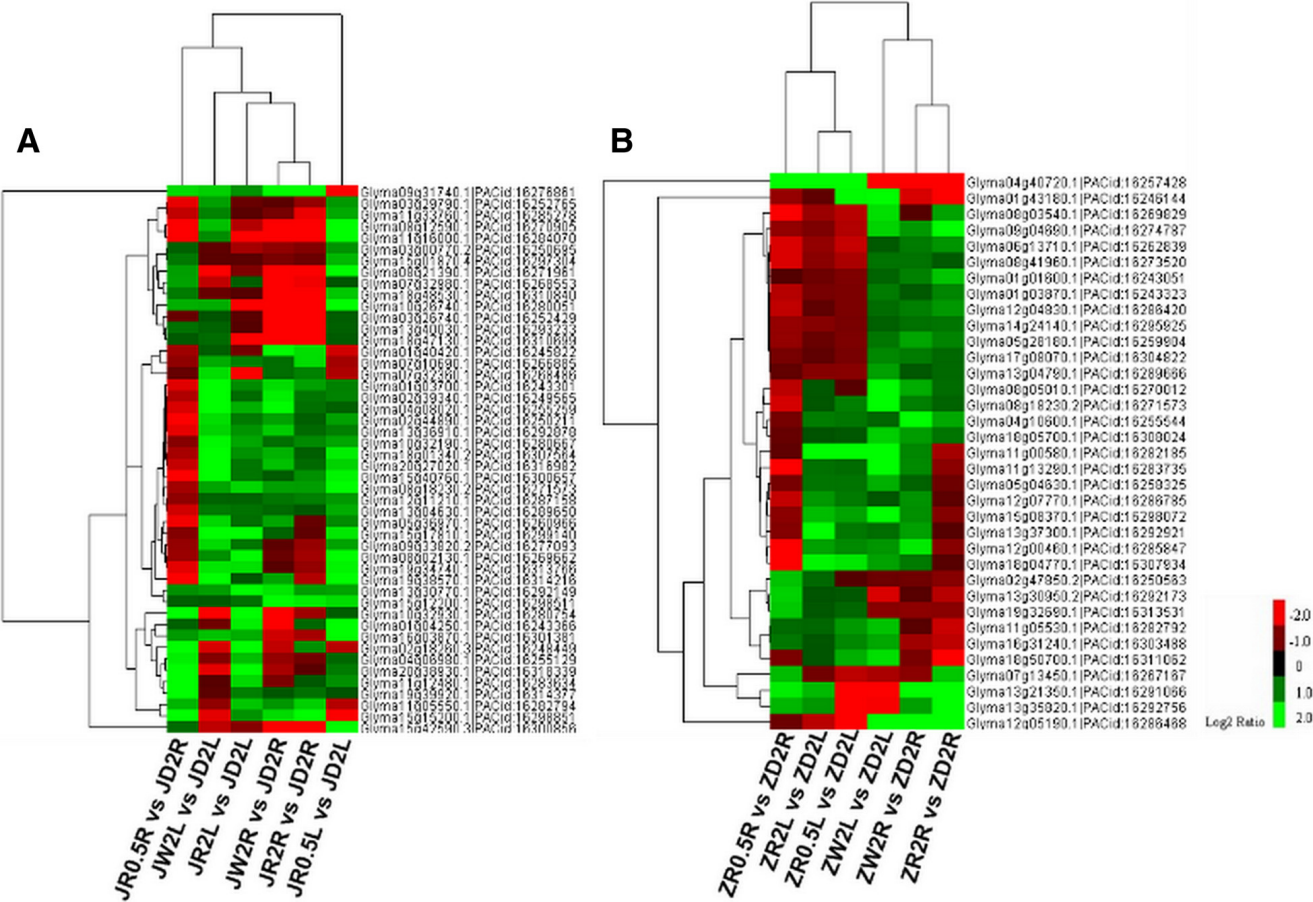
**Hierarchical clustering and heat map presentation of DEGs in two genotypes under dehydration and rehydration.** The vertical dendrogram indicates the relationship among transcripts across tissues and treatments in the hierarchical cluster analysis. Each column represents an experimental condition and each row represents a gene. Expression differences are shown in different colors. Red indicates down-regulation and green indicates up-regulation. **(A)** Jindou21*.***(B)** Zhongdou33*.*

Genes with similar expression patterns are usually functionally correlated. We performed cluster analysis of gene expression patterns using Cluster and Java TreeView software [39,40]. The results of hierarchical cluster analysis of DEGs under dehydration and rehydration treatments are shown in Figure 8. In the drought-tolerant genotype, 48 genes were identified between (JW2L vs. JD2L)∩(JR0.5 L vs. JD2L)∩(JR2L vs. JD2L)∩(JW2R vs. JD2R)∩(JR0.5R vs. JD2R)∩(JR2R vs. JD2R); 35 genes were found between (ZW2L vs. ZD2L)∩(ZR0.5 L vs. ZD2L)∩(ZR2L vs. ZD2L)∩(ZW2R vs. ZD2R)∩(ZR0.5R vs. ZD2R)∩(ZR2R vs. ZD2R) in the drought-sensitive genotype. Under rehydration conditions, some drought-inducible genes (Glyma03g29790, Glyma11g33760, Glyma08g12590, Glyma11g16000, and Glyma01g43180) were down-regulated, while drought-repressed genes (Glyma11g05550, Glyma15g15200, Glyma13g21350, and Glyma13g35820) were up-regulated upon re-watering. These genes may facilitate adaptation to drought conditions and promote plant growth recovery upon rewatering.

### Candidate DEGs for major roles in response to dehydration stress

The seven most differentially expressed genes (|log_2_ ratio| ≥ 8) (Glyma15g03920, Glyma05g02470, Glyma15g15010, Glyma05g09070, Glyma06g35630, Glyma08g12590, and Glyma11g16000) identified by analysis of DEGs in the drought-tolerant genotype and by cluster analysis warrant further study. Functional annotations of these genes are given in Tables 2, 3, and 4. Functional analysis of these genes should assist efforts to improve soybean drought tolerance.

### Evaluation of housekeeping gene stability and verification of DGE results by QRT-PCR

The software package geNorm was used to evaluate the stability of expression of housekeeping genes. UKN2 and HDC were ranked the most stable in all samples in our experiment, while TUA5 and UBQ10 consistently ranked poorly. Optimal numbers of housekeeping genes required for RT-PCR data normalization were then determined by geNorm (Additional file 12); using this program, three housekeeping genes (HDC, UKN2, and ACT2/7) were selected to normalize gene expression levels (V3/4 = 0.122 < 0.15) (Additional file 13).

**Figure 9:**
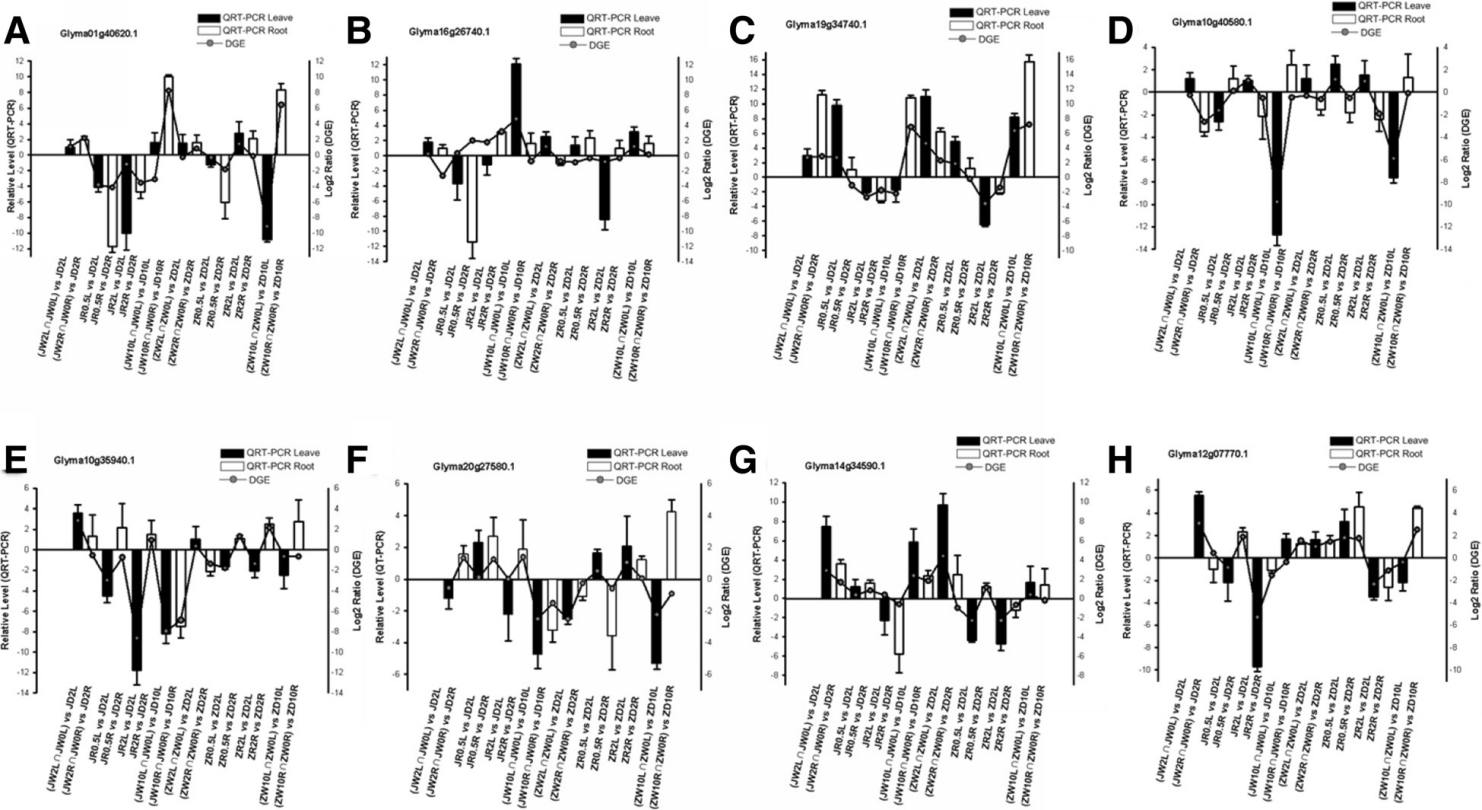
**Results of QRT-PCR on eight genes in leaves and roots under dehydration and rehydration treatments. (A)** Glyma01g40620.1. **(B)** Glyma16g26740.1. **(C)** Glyma19g34740.1. **(D)** Glyma10g40580.1. **(E)** Glyma10g35940.1. **(F)** Glyma20g27580.1. **(G)** Glyma14g34590.1. **(H)** Glyma12g07770.1.

To confirm DGE results, QRT-PCR was conducted on eight randomly selected DEGs based on transcriptional profile analysis (Figure 9). QRT-PCR results agreed with the transcriptional profile data for 96 out of 128 (75%) data points. Although specific expression values obtained using QRT-PCR were not exactly identical to fold changes calculated from expression profiles, both methods yielded identical expression trends. QRT-PCR results ultimately reflected consistency with the transcriptional profile data. Sequences of specific primers used for QRT-PCR are given in Additional file 3.

## Discussion

In this study, DGE technology, which allows acquisition of more DEGs and related biological information that microarray-based techniques [41,42], was used to identify DEGs of soybean under dehydration and rehydration conditions. This is the first reported attempt to identify soybean dehydration-responsive TFs, PKs, and other regulatory proteins using both drought-tolerant and drought-sensitive genotypes. Although identification of DEGs in Arabidopsis, rice, and other plants has been reported [43-45], there have been few studies in soybean. The acquisition of soybean candidate genes would aid the study of soybean drought-tolerant molecular mechanisms and, more broadly, drought stress responses in dicotyledonous crop plants.

The drought strategy employed here was similar to a previously used air-drying method, where rapid dehydration was adopted to simulate drought conditions [46]. In our experiments, phenotypes exhibited by soybean in response to drought stress mainly involved leaf wilting and rolling; leaf etiolation was not observed. During screening for drought-tolerant genotypes, we discovered that RWC and REC varied significantly, but chlorophyll content and photosynthetic rate did not (data not shown). We believe that a decrease in water content due to stomatal closure, the primary symptoms of which are leaf wilting and rolling, was caused by the rapid dehydration; in contrast, there was no change in chlorophyll content and photosynthetic rate in the seedlings in response to short-term water deficit. A future search for candidate genes related to these physiological indices will incorporate long-term drought stress conditions.

There is much evidence that root and/or leaf traits are closely related to mechanisms of drought tolerance [16]. Root morphology and development is one of the most important factors correlated with mechanisms of drought tolerance [47]. The relationship between roots and leaves during drought stress is controversial. In one study, it was proposed that roots, which are more sensitive to changes in soil environment, could send drought stress signals to leaves [48]. On the other hand, other researchers have pointed out that leaf growth restraint can aid survival during drought by minimizing the evaporative leaf surface area [49]. In our study, soybean leaves and roots were thus collected separately for sequencing.

Because analysis of DEGs under re-watering is important for elucidation of molecular mechanisms during the recovery process from dehydration to rehydration [44], DEGs were identified during the rehydration process in our study in addition to dehydration. We found that some drought-inducible genes were repressed under re-watering treatment, while the expression of genes down-regulated under dehydration was promoted during rehydration.

Most of the genes differentially expressed during rehydration could not be annotated. Several genes that were functionally annotated encode MYB and homeodomain-like TFs and may play central roles in plant response to dehydration and growth recovery; however, their functions require further study.

### GO functional annotation of DEGs

In addition to their identification, DEGs were annotated according to biological process using GO functional enrichment analysis. The primary enriched biological processes associated with DEGs in the drought-tolerant genotype were metabolic processes, response to stress, response to stimulus, transcription, hormone-mediated signaling pathways, regulation of gene expression, protein modification processes, and plant-type cell wall biogenesis (Additional file 10). A large proportion of metabolic processes were related to carbohydrate metabolism, which could provide most of the energy required for these pathways under dehydration. Other biological process categories enriched in DEGs included aspartate family amino acid and Acetyl-CoA metabolic processes (Glyma14g37440.1, Glyma11g27720.2 and Glyma15g15010.1) that also contribute to energy release [50]. Proline is an important osmolyte, with its synthesis derived from glutamine metabolic processes; consequently, genes associated with glutamine family amino acid and proline metabolisms (Glyma14g13480.2, Glyma11g27720.2, Glyma14g37440.1, and Glyma13g07110.1) are necessary for response to osmotic stress [51]. Dicarboxylic acid metabolism (Glyma14g13480.2) is also reportedly involved in drought stress [52]. In addition, two regulatory proteins (Glyma01g01190.1 and Glyma15g03920.1) were identified in the tolerant genotype. These proteins may protect plants by promoting the formation of a tolerant morphological structure to reduce the impact of adversity [53]. In addition to genes associated with metabolic processes and morphogenesis, DEGs involved in other processes were mainly regulatory proteins, such as transcription factors and protein kinases.

Transcription factors and regulatory proteins are important classes of genes that may regulate expression of downstream drought-responsive genes [54,55]. Understanding the functions, signaling, and metabolic pathways of TFs, PKs, and other regulatory proteins under dehydration will help reveal drought response networks in soybean.

### Dehydration stress-inducible TF and PK families in soybean

Transcription factors are known to function in the regulation of target gene expression. A single transcription factor can regulate the expression of multiple genes. Transcriptional control of stress-response gene expression is a crucial component of plant response to a range of environmental stresses [56]. Several transcription factors have been reported to be related to drought tolerance in plants. In our study, many DEGs encoding TF families were identified in the tolerant genotype (Table 2). Among these TFs, MYB TFs, HD-ZIP TFS, AP2/EREBP TFs, and zinc finger proteins play a central role in drought tolerance [57-60]. Ring finger proteins, bHLH proteins, and bZIP TFs regulate the stress-responsive ABA signaling pathway [61,62]. HSFs are key regulators in the induction of defense systems under various stresses [63]. In addition, ARFs and ERFs are also involved in stress responses [64,65]. Many members of the PK family, which plays a key role in signal transduction [66,67], were also identified in the tolerant genotype (Table 3). Among these, MAPKs are critical signal transduction factors. They can modulate the interaction of defense pathways activated by biotic (pathogen infection) and abiotic (wounding, drought, salt, and cold) factors [68]. MAPKs and S/T PKs can increase multistress tolerance [69,70]. CDPKs mainly function in the ABA signal pathway and Ca^2+^-mediated regulation [67,71], and RPKs enhance abiotic stress in Arabidopsis and rice [72,73]. We thus believe that the presence of these TFs and PKs detected by DGE profiling indicates that various signal molecules act to improve drought tolerance in soybean.

### Other dehydration stress-inducible regulatory proteins in soybean

In addition to TFs and PKs, some regulatory proteins related to plant growth, development, and metabolism were detected in this study (Table 4). It has been reported that ATP synthesis during drought stress is essential for preserving plastid function [74]. Among our DEGs, some ATP-binding proteins identified in the tolerant genotype were down-regulated, which might make more ATP available for maintenance of cellular activities under water deficit. Cytochrome P450s are involved in ABA catabolism in drought stress response [75]. They can reduce and eliminate reactive oxygen damage, serve as precursors to ABA synthesis, and also participate in photosynthesis. In our study, the expression of several identified cytochrome P450s was suppressed. Mechanisms involving two-component gene systems play important roles in cellular stress signal transduction [22]. A two-component histidine kinase 1 (ATHK1) is reported to function as an osmosensor, and phospholipase C (PLC) is thought to function upstream of the drought-responsive DREB2 system [76]. In our study, four two-component system genes were identified. Two genes separately encoding histone and aspartate aminotransferase were down-regulated, and another gene encoding asparagine synthetase was up-regulated. Regulatory factors of two-component systems are induced by drought, salt, and osmotic stress [77,78]. These identified DEGs thus likely play an important role in improving drought tolerance.

Based on pathway enrichment analysis of the DEGs using KEGG, the major public pathway-related database [79], genes identified in the tolerant genotype are mainly involved in signal transduction and metabolic pathways.

### Signal transduction pathways related to TF and PK regulation

Consistent with our results, a key gene encoding indole-3-acetaldehyde oxidase has been identified in tomato under water deficit [45]. In our study, we detected seven genes involved in auxin/IAA pathways (Table 2). They are downstream genes in the tryptophan metabolic pathway, possibly associated with auxin/IAA synthesis in response to water deficit. In addition, two genes encoding MYC proteins were found to be involved in the jasmonic acid signal pathway. The two genes are located downstream of the α-linolenic acid metabolic pathway. Three ethylene-responsive factors, involved in downstream regulation of cysteine and methionine metabolism, were identified as well. Jasmonic acid and ethylene are factors involved in response to senescence stress [80,81]. We therefore speculate that these five genes encoding MYC proteins and ethylene-responsive factors may regulate leaf senescence in response to water deficit. With respect to the plant-pathogen interaction pathway, identified genes included some encoding MYC proteins and PKs. MYC proteins are also involved in the jasmonic acid signaling pathway, and may be key nodes between the two pathways. Protein kinases mainly exist in cell membranes (FLS_2_ and BAKIFKK1) and the cytoplasm (CDPK, RPM1, and MEKK), and may possibly regulate induction of downstream defense-related genes such as WRKYs. The stomatal closure signaling pathway, regulated by ABA, and the immune pathway, dependent on FLS_2_ and MEKK1, are interconnected [82,83]. In our study, we not only detected proteins related to response to ABA, but also identified FLS_2_ and MEKK1 proteins associated with the plant defense pathway. Consequently, we suggest that under dehydration these pathogen-interaction proteins may increase plant defense abilities in addition to enhancing drought tolerance.

### HSP and regulatory enzyme involvement in developmental processes and metabolic pathways

The results of our DGE analysis indicate that some HSP proteins and key enzymes may play roles in regulating soybean growth and development during dehydration. HSP genes are molecular chaperones involved in a variety of cellular processes, including protein folding, protein transport across membranes, regulation of protein degradation, and prevention of irreversible protein aggregation. HSP over-expression increases drought tolerance in transgenic rice and tobacco [84,85]. The drought stress response functions of HSP genes identified in our study require further validation. Three protein enzymes identified in our study—pyruvate dehydrogenase, pyruvate kinase, and tyrosine aminotransferase—may be involved in the glycolysis pathway contributing to the release of energy to maintain plant normal metabolism under water deficit.

## Conclusions

This study has demonstrated the usefulness of the DGE approach for identification of DEGs between two soybean genotypes under dehydration and rehydration conditions. Based on these results, seven candidate dehydration-responsive DEGs were selected for further study. Future work concentrating on their functions under drought stress may lead to a better understanding of genetic mechanisms underlying phenotypic differences between the two soybean genotypes under dehydration conditions. Elucidation of such mechanisms is required for the improvement of soybean drought tolerance.

## Abbreviations

ABA: Abscisic acid; ARFs: Auxin-related factors; CDPKs: Calcium-dependent protein kinases; DEGs: Differentially expressed genes; DGE: Digital Gene Expression Tag profile; ERFs: Ethylene-related factors; HSFs: Heat shock factors; MAPKs: Mitogen-activated protein kinases; PKs: Protein kinases; QRT-PCR: Quantitative real-time PCR; REC: Relative electrical conductivity; RPKs: Receptor protein kinases; RWC: Relative water content; S/T PKs: Serine/threonine protein kinases; TFs: Transcription factors; HSP: Heat shock protein; MEKK: MEK kinase; FLS2: Flagellin sensing2; RPM1: RESISTANCE TO P. SYRINGAE PV MACULICOLA 1.

## Competing interests

The authors declare that they have no competing interests.

## Authors’ contributions

XAZ and WBL conceived the experiments, which LMC designed and performed. Data analysis was conducted by LMC and WC; RZ provided the soybean seeds used for research. LMC, CW, AHS, ZHS, CJZ, DZQ, ZLY, and SLC supplied reagents, materials, and analytical tools. LMC wrote the manuscript. All authors read and approved the final manuscript.

## Supplementary Material

Additional file 1 **List of screened soybean material.** Fifty-seven soybean genotypes from different areas of China were used in preliminary screening under different dehydration times, from which two drought-tolerant genotypes and two drought-sensitive genotypes were selected. Click here for file

Additional file 2 Tag preparation principles and steps, including cDNA synthesis, enzyme digestion, adaptor ligation, and sequencing. Click here for file

Additional file 3 **Primers used in the QRT-PCR analysis.** Primers listed were used to amplify eight genes that were randomly selected for QRT-PCR assays to confirm DEG reliability and five housekeeping genes used to quantify gene expression. Click here for file

Additional file 4 **Categorization and abundance of tags.** The number of total tags and distinct tags calculated for different tag abundance categories from 28 libraries. Click here for file

Additional file 5 **Sequencing saturation analysis of 28 libraries.** The number of detected genes was found to increase as the total number of tags increased. Click here for file

Additional file 6 **Genes responsive to dehydration only in tolerant-genotype leaves.** DEGs between tolerant and sensitive genotypes were obtained by bioinformatics analysis; DEGs expressed under drought only in the tolerant-genotype leaves are listed. Click here for file

Additional file 7 **Genes responsive to dehydration only in tolerant-genotype roots.** DEGs between tolerant and sensitive genotypes were obtained by bioinformatics analysis; DEGs expressed under dehydration only in the tolerant genotype roots are listed. Click here for file

Additional file 8 **Genes responsive to rehydration after dehydration only in tolerant-genotype leaves.** DEGs between tolerant and sensitive genotypes were obtained by bioinformatics analysis; DEGs expressed under re-watering only in tolerant-genotype leaves are listed. Click here for file

Additional file 9 **Genes responsive to rehydration after dehydration only in tolerant-genotype roots.** DEGs between tolerant and sensitive genotypes were obtained by bioinformatics analysis; DEGs expressed under re-watering only in tolerant-genotype roots are listed. Click here for file

Additional file 10 **Gene Ontology functional annotation of DEGs in the drought- tolerant genotype.** The most significantly enriched GO biological processes and their associated DEGs are listed. Click here for file

Additional file 11 **Pathway enrichment analysis of DEGs.** Pathway enrichment analyses of DEGs are summarized. Click here for file

Additional file 12 **Evaluation of housekeeping gene expression stability using geNorm software.** Stabilities of TUA, UBQ, ACT2/7, UKN2, and HDC housekeeping genes were analyzed. Click here for file

Additional file 13 **Standardized calculation of a selected gene.** The expression level of one randomly chosen gene was compared between DGE and QRT-PCR results. Click here for file
