# Supplementary material for: Genome-wide transcriptional analysis of two soybean genotypes under dehydration and rehydration conditions
Source: BMC Genomics. 2013 Oct 6;14:687. doi: 10.1186/1471-2164-14-687 (PMC3827939; doi:10.1186/1471-2164-14-687)
Supplement: Additional file 2 — Tag preparation principles and steps, including cDNA synthesis, enzyme digestion, adaptor ligation, and sequencing. [file 1471-2164-14-687-S2.doc]

**Specified Experimental Process of Raw Sequences Production:**

Extract 6 µg total RNA, use Oligo(dT) magnetic beads adsorption to purify mRNA, and then use Oligo(dT) as primer to synthesize the first and second-strand cDNA. The 5' ends of tags can be generated by two types of endonuclease, NlaIII or DpnII. Usually, the bead-bound cDNA is subsequently digested with restriction enzyme NlaIII, which recognizes and cuts off the CATG sites. The fragments apart from the 3' cDNA fragments connected to Oligo(dT) beads are washed away and the Illumina adaptor 1 is ligated to the sticky 5' end of the digested bead-bound cDNA fragments. The junction of Illumina adaptor 1 and CATG site is the recognition site of MmeI, which is a type of Endonuclease with separated recognition sites and digestion sites. It cuts at 17bp downstream of the CATG site, producing tags with adaptor 1. After removing 3' fragments with magnetic beads precipitation, Illumina adaptor 2 is ligated to the 3' ends of tags, acquiring tags with different adaptors of both ends to form a tag library. After 15 cycles of linear PCR amplification, 105bp fragments are purified by 6% TBE PAGE Gel electrophoresis. After denaturation, the single-chain molecules are fixed onto the Illumina Sequencing Chip (flowcell). Each molecule grows into a single-molecule cluster sequencing template through Situ amplification. Then add in four types of nucleotides which are labeled by four colors, and perform sequencing with the method of sequencing by synthesis (SBS). Each tunnel will generate millions of raw reads with sequencing length of 49bp.


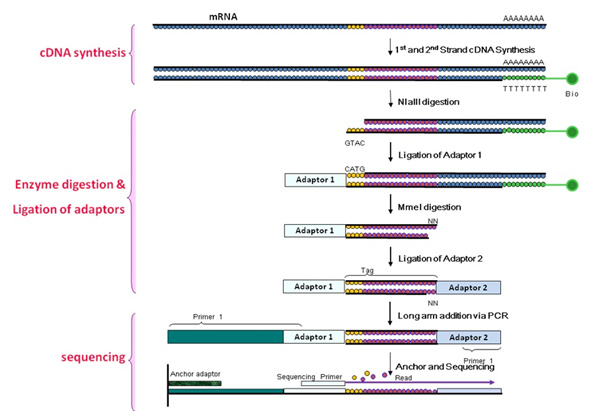


**Additional file 2**. The principle and step of tag preparation
