# Supplementary material for: Genome-wide transcriptional analysis of two soybean genotypes under dehydration and rehydration conditions
Source: BMC Genomics. 2013 Oct 6;14:687. doi: 10.1186/1471-2164-14-687 (PMC3827939; doi:10.1186/1471-2164-14-687)
Supplement: Additional file 5 — Sequencing saturation analysis of 28 libraries. The number of detected genes was found to increase as the total number of tags increased. [file 1471-2164-14-687-S5.doc]

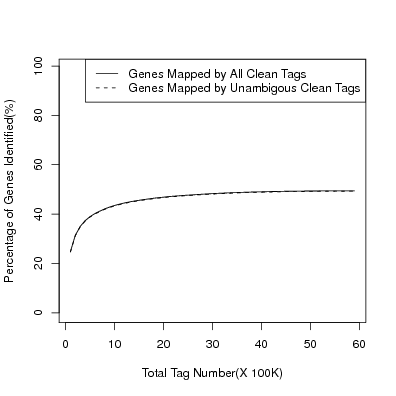

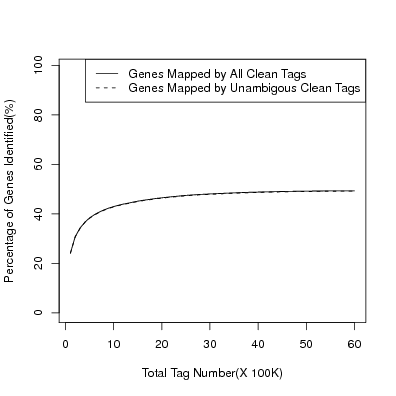


JW0L

JW0R


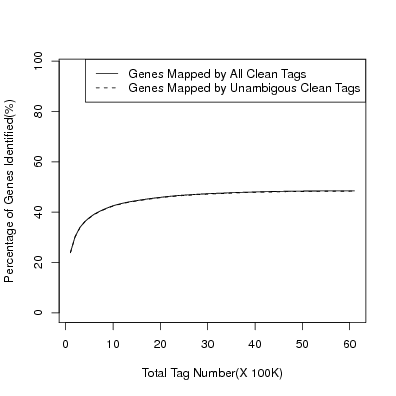

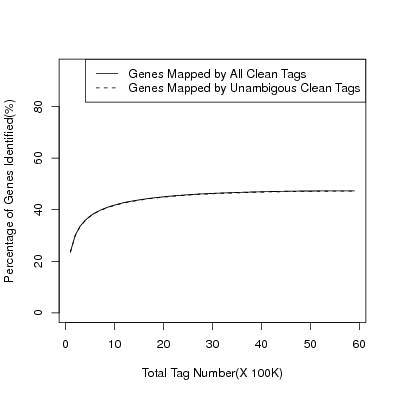


JD2L

JW2L


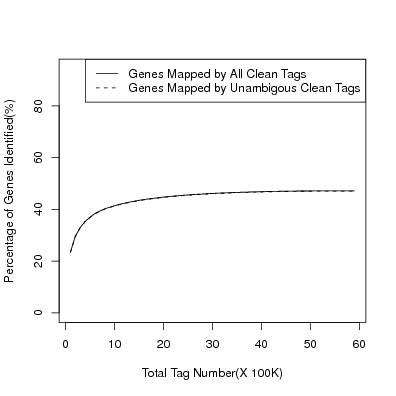

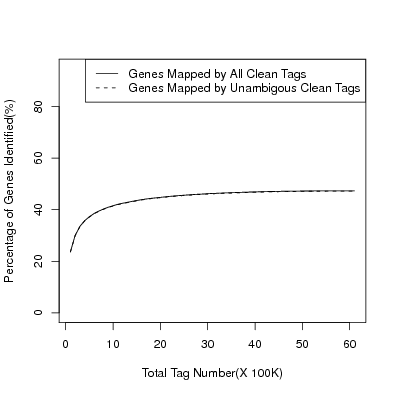


JD2R

JW2R


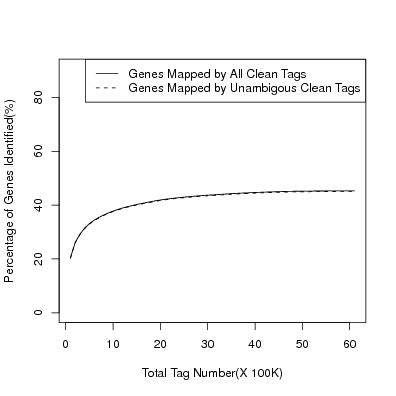

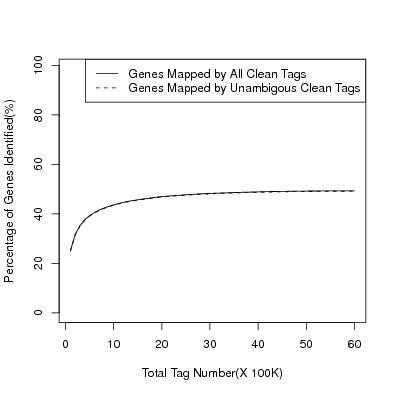


JD10L

JW10L


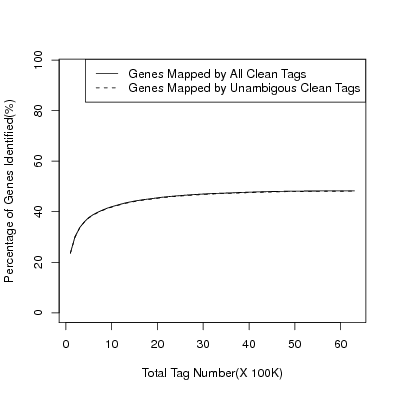

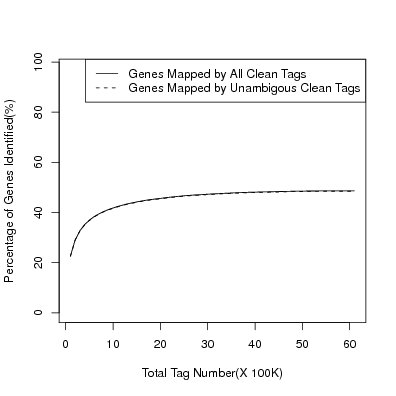


JD10R

JW10R


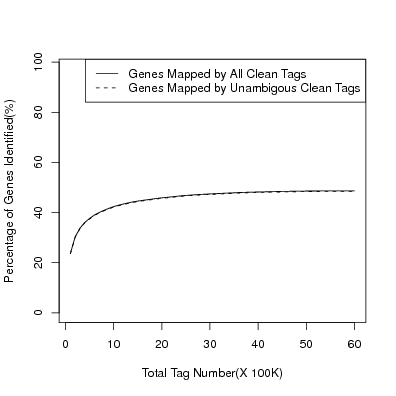

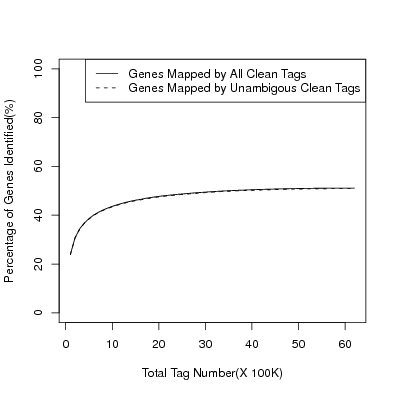


ZW0L

ZW0R


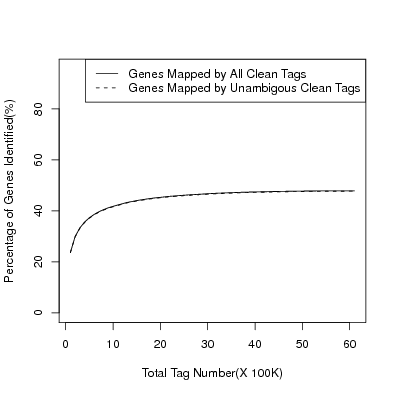

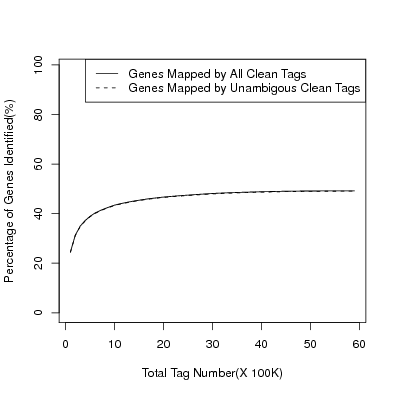


ZD2L

ZW2L


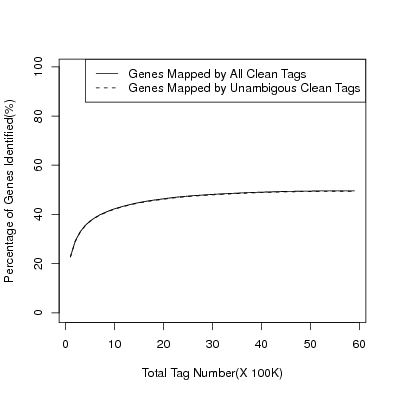

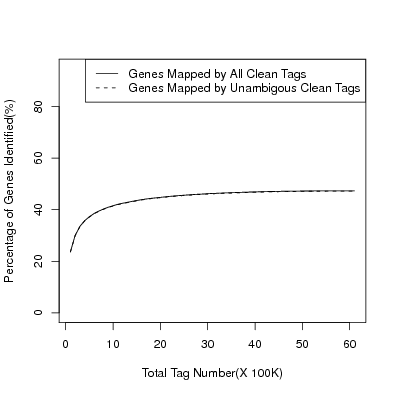


ZD2R

ZW2R


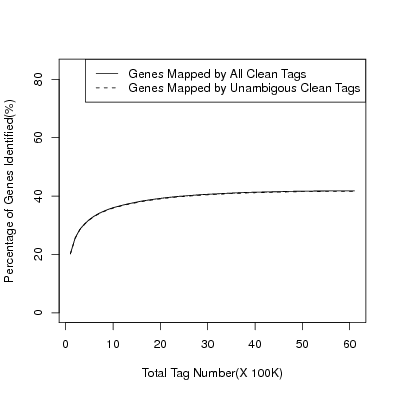

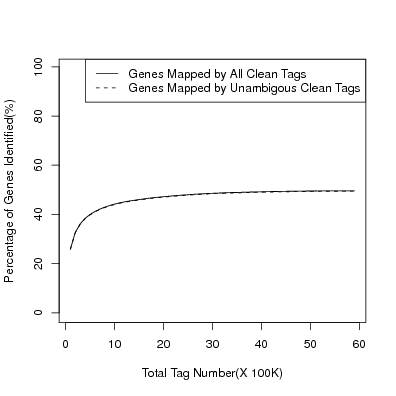


ZD10L

ZW10L


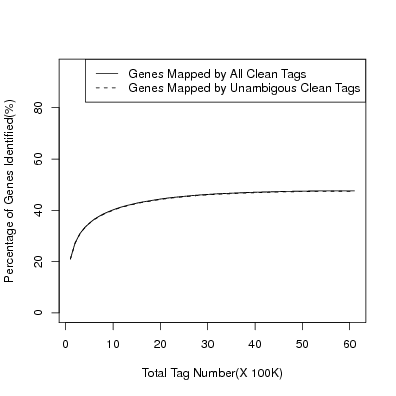

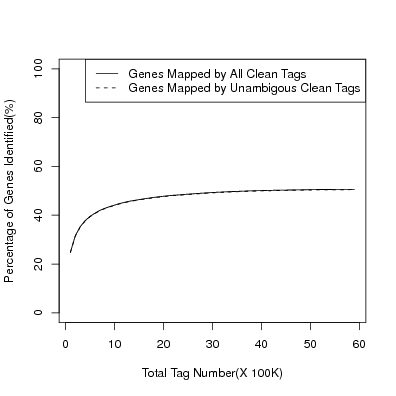


ZD10R

ZW10R


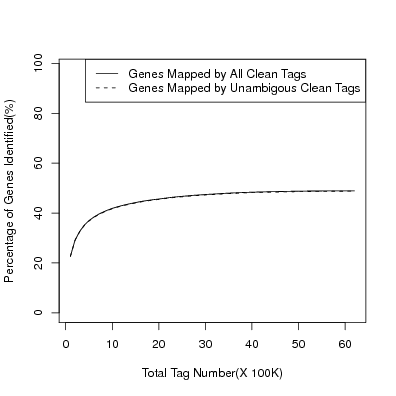

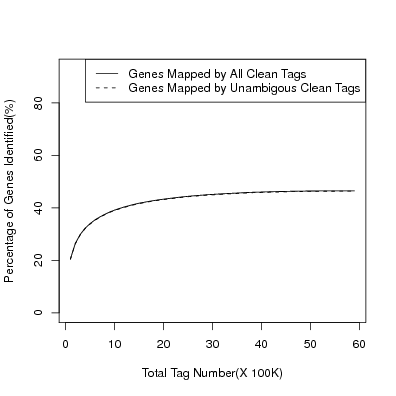


JR0.5L

JR0.5R


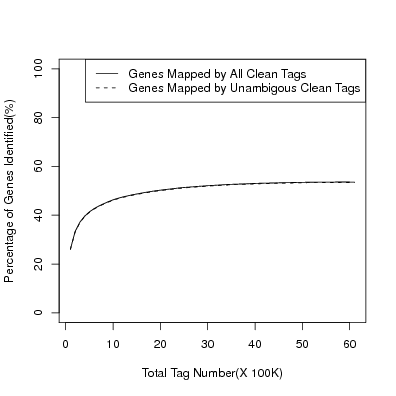

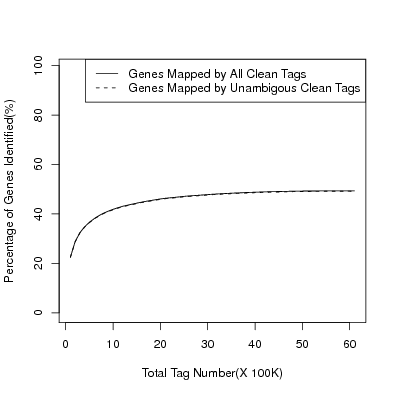


JR2L

JR2R


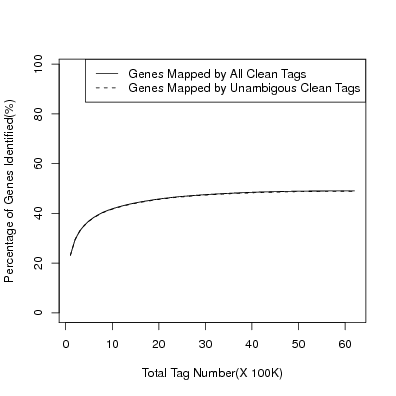

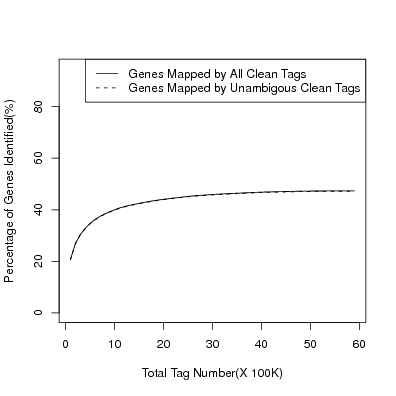


ZR0.5L

ZR0.5R


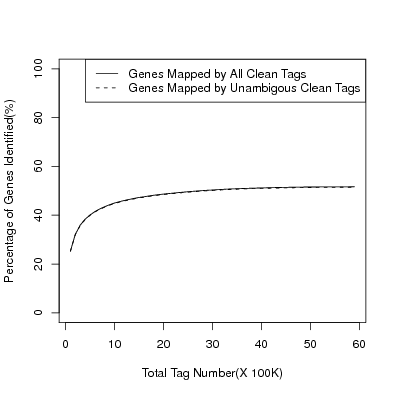

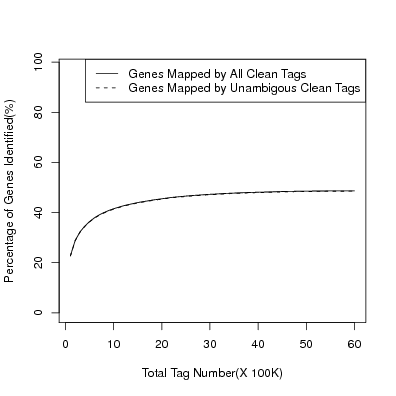


ZR2L

ZR2R

Additional file 5. Sequencing saturation analysis of the twenty-eight libraries. The number of detected genes was found to increase as the total number of tags increased.
